# Assessment of the German Version of Brief Assessment of Cognition in Schizophrenia (BACS)

**DOI:** 10.1016/j.scog.2025.100364

**Published:** 2025-04-30

**Authors:** Matin Mortazavi, Jakob Amon, Iris Jäger, Genc Hasanaj, Zahra Aminifarsani, Kristin Fischer, Matthias Gamer, CDP Working Group, Alkomiet Hasan, Richard S.E. Keefe, Gabriele Sachs, Peter Falkai, Daniel Keeser, Florian Raabe, Elias Wagner

**Affiliations:** a Department of Psychiatry, Psychotherapy and Psychosomatics, Medical Faculty, University of Augsburg, BKH Augsburg, Augsburg, Germany; b Evidence-based Psychiatry and Psychotherapy, Faculty of Medicine, University of Augsburg, Augsburg, Germany; c Department of Psychology (Experimental Clinical Psychology), University of Würzburg, Würzburg, Germany; d German Center for Mental Health (DZPG), partner site Munich/Augsburg, Germany; e Duke University, Durham, NC, USA; f Medical University of Vienna, 1090 Vienna, Austria; g Department of Psychiatry and Psychotherapy, LMU University Hospital, LMU Munich, Germany; h Max Planck Institute of Psychiatry, Munich, Germany

**Keywords:** Schizophrenia, Cognition

## Abstract

**Background:**

Cognitive impairments are a hallmark of schizophrenia-spectrum disorders (SSD), contributing to poor treatment outcomes and a key treatment target. The Brief Assessment of Cognition in Schizophrenia (BACS) battery is a validated tool designed to evaluate affected core domains in SSD. The present study evaluated psychometric properties of the German version of the BACS in a representative sample of individuals with SSD and healthy control subjects.

**Methods:**

*N* = 107 individuals with SSD and *n* = 175 healthy controls were assessed with the German version of the BACS. Diagnosis was confirmed with the Mini International Neuropsychiatric Interview according to DSM-V. Validity was assessed through pair-wise comparisons between SSD individuals and healthy controls and by using receiver operating characteristic analysis. Internal consistency as a measure of reliability was evaluated using McDonald's Omega and Cronbach's Alpha in addition to factor and principal component analysis.

**Results:**

All individuals with SSD exhibited significantly lower z-scores across all BACS subtests and BACS composite scores (Z < -1.5) compared to healthy controls. ROC analysis revealed good diagnostic accuracy with an AUC of 0.83 (95%CI: 0.78,0.88, sensitivity = 0.75, specificity = 0.75). Similar results were observed in sub-cohorts comprising clinically stable SSD patients and those with younger ages (18–35 years old). A unidimensional structure, supported by McDonald's Omega (ω = 0.72) and principal component analysis, confirmed robust internal reliability.

**Conclusions:**

The German BACS demonstrates strong validity and internal reliability when assessed in a representative case-control sample. This study provides an extensive normative dataset for individuals with SSD in German-speaking populations, facilitating future research and clinical assessments of cognition.

## 1 Introduction

Cognitive impairments are a hallmark of schizophrenia-spectrum disorders (SSD) and contribute substantially to poor social prognosis, poor treatment outcomes (McCutcheon et al., 2023; Kahn and Keefe, 2013) and indirect costs of the disease (McEvoy, 2007). Cognitive impairment is typically present before onset of psychotic symptoms, varies markedly in affected individuals and currently no effective pharmacological treatment option is available to prevent or arrest the cognitive impairment associated with schizophrenia (CIAS) (McCutcheon et al., 2023; Vita et al., 2022; Nuechterlein et al., 2024). Individuals with SSD are exposed to an increased risk to develop an earlier onset of cognitive decline in later life compared to healthy individuals presumably due to neurovascular factors (McCutcheon et al., 2023). To disentangle the complex pathophysiological mechanisms behind CIAS was once named the holy grail of schizophrenia research (Kahn and Keefe, 2013) and the neural pathways with presumed pivotal contribution to dyscognitive symptoms in SSDs are at the center of focus in phase II/III randomized controlled trials (Fleischhacker et al., 2021). It is known that individuals with SSD are significantly impaired in their overall cognitive performance, which, on average, is estimated to be two standard deviations below that of healthy controls (Keefe et al., 2011) and that the extent of CIAS is a strong predictor of long-term functional impairment for individuals with SSD (Green, 2006).

The Brief Assessment of Cognition in Schizophrenia (BACS) is a reliable and valid test battery that evaluates various aspects of cognition which are typically impaired in individuals with SSD. The BACS encompasses four of the most important cognitive domains, which are typically the focus of psychopharmaceutical research in the domain of cognition in SSD, i.e. reasoning and problem-solving, processing speed, verbal memory, and working memory (Kaneda et al., 2013). Keefe et al. (Keefe et al., 2004; Keefe et al., 2008) standardized the original English version of the BACS and collected normative data from 404 healthy controls. Composite scores of the BACS correlate with functional capacity and real-world functioning (Keefe et al., 2006) and the US Food and Drug Administration indicated that substantial improvement on a consensus cognitive performance endpoint, such as the BACS, together with a co-primary measure of functioning would be crucial for drug approval of cognition-enhancing drugs for schizophrenia (Harvey et al., 2022). In recent years, the BACS has been translated and standardized for several languages across the world, paving the way for its broader utilization and extending BACS normative data to different languages. The psychometric properties of the German version of BACS was evaluated in a preliminary study by Sachs et al. using a small sample of *n* = 30 people diagnosed with schizophrenia according to DSM-IV and n = 30 healthy controls (Sachs et al., 2011). While their results showed a good degree of validity and reliability for German BACS, the underpowered sample size does not seem to represent the wide spectrum of clinical phenotypes in SSD and may not serve as normative data for future large-scale studies examining BACS outcomes in German speaking SSD populations.

The present study employed the largest available case-control cognitive dataset to date in German-speaking people with SSD and aimed to replicate and extend the findings of Sachs et al. (Sachs et al., 2011) by evaluating certain psychometric properties of the German BACS and provide new normative data for the German version of the BACS.

## 2 Methods

### 2.1 Subjects

Diagnosis was confirmed using the German version of Mini International Neuropsychiatric Interview (M.I.N.I. German version 7.0.2) (Sheehan et al., 1998; Ackenheil et al., 1999). Healthy controls were defined as participants with no neuropsychiatric disorder according to DSM-V criteria. The sample was collected as part of the cross-diagnostic Munich multimodal clinical deep phenotyping study (Krčmář et al., 2023), which was approved by the local ethics committee at the Ludwig-Maximilian-University of Munich, Germany (project number 20–528). The study is registered in the German Clinical Trials Register (ID: DRKS00024177). Inclusion criteria were as follows: age between 18 and 65 years, no diagnosed brain disorders (i.e. multiple sclerosis, stroke), no intake of lorazepam ≥2 mg or diazepam ≥20 mg as well as no current electroconvulsive therapy in patients at time of the assessment due to potential interference with BACS performance. Individuals with a primary psychiatric diagnosis other than SSD were excluded. Circumstances which could systematically affect BACS performance (e.g. language barrier, perceived sedated state, training effects) led to exclusion. All participants gave their written informed consent. Importantly, patients with a score below 63 on the Positive and Negative Syndrome Scale (PANSS) (Kay et al., 1987), comparable to approximately the upper limit of a score of 3 (“mildly ill”) on the Clinical Global Impressions Symptom scale (CGI-S) (Levine et al., 2008) were defined as clinically stable. Table 1 provides demographical and clinical characteristics of the final sample. Furthermore, another subgroup was formed consisting of individuals with an age range of 18–35 years corresponding to that of Sachs et al. (Sachs et al., 2011) for better comparability of our findings.

**Table 1:** Demographic and clinical features of study cohorts.

	Clinically stable SSD individuals		All SSD individuals		Healthy subjects	
	Mean	SD	Mean	SD	Mean	SD
N	92		107		175	
Age	39.77	11.36	39.7	11.42	33.95	12.65
Sex (n, female/male)	38/54		42/65		95/80	
BMI (in kg/m^2^)	29.26	6.84	29.04	6.69	18.05	2.27
Chlorpromazine equivalent (in mg)	292.13	224.50	296.45	231.54		
PANSS positive score	10.58	3.26	11.58	4.21	7.27	0.7
PANSS negative score	10.24	3.63	11.30	4.70	7.39	0.88
PANSS general score	23.59	5.13	25.44	6.95	16.78	1.4
PANSS total score	44.30	8.83	48.23	13.11	31.44	2.31
GAF	59.28	9.46	57.74	10.31	90.44	5.98
CDSS score	2.38	1.97	2.50	1.93	0.29	0.7
Level of education (in years)	17.02	5.78	16.23	5.52	16.36	6.07
Age at first symptoms (in years)	26.29	9.73	27.04	10.22		
Disease duration	12.45	8.82	11.88	8.83		
Number of hospitalizations	4.10	3.48	4.20	3.58		
Lifetime clozapine treatment (n)	18		21			
First-episode psychosis (n)	9		13			
Schizophrenia/schizoaffective diagnosis (n)	88		100			

**Abbreviations:** CDSS: Calgary Depression Scale for Schizophrenia; BMI: Body Mass Index; PANSS: Positive and Negative Syndrome Scale, GAF: Global Assessment of Functioning.

### 2.2 German BACS and clinical assessment

The German version of BACS battery was used to evaluate cognition in all participants and carried out by trained study personnel (Sachs et al., 2011). Our BACS battery encompassed the six subtests for the assessed cognitive domains, namely verbal memory, fluency, attention, speed of processing, working memory, motor speed, and executive abilities (Keefe et al., 2004). If a subset was completed by a subject, their score was added to the pool and entered the analysis for that subset. The BACS composite score was only calculated and later considered in analysis for subjects who completed all six BACS subtests. Details of the clinical assessments performed in this study are outlined in our published study protocol and previous publications (Krčmář et al., 2023; Boudriot et al., 2023). Briefly stated, severity of SSD symptoms was assessed using the PANSS (Kay et al., 1987) and depression was assessed by the Calgary Depression Scale for Schizophrenia (CDSS) (Addington et al., 1993). Moreover, the following information were collected for individuals with SSD: medication history, age at first SSD symptoms and number of hospitalizations. In all individuals, age, sex, education level, somatic comorbidities (e.g. hypertension, diabetes) and body-mass-index (BMI) were assessed. Level of education was an arithmetic sum of self-reported years of schooling and years of vocational training. Previous medical records were reviewed when provided. Dosage of current antipsychotic medications were converted to chlorpromazine equivalents using the Defined Daily Dose method (Leucht et al., 2020). Lifetime clozapine treatment was used as a proxy for pharmacological treatment-resistance (Ruderfer et al., 2016).

### 2.3 Statistical analysis

Normative data was provided following the procedure used by Keefe et al. (Keefe et al., 2004) where the primary measure of each subtest of the BACS is standardized by creating z-scores. The mean of the healthy control group is set to 0 and the standard deviation is set to 1. A composite score is then calculated by averaging the six standardized measures for those subjects who completed all BACS subtests. A new z-score is then calculated for the composite score. To test for homogeneity of variance, we used the Levene test. If homogeneity of variance was not given, we used the Welch test to perform pair-wise comparisons. Otherwise, the *t*-test for independent samples was calculated. Correlation matrices were computed using the Pearson correlation coefficient.

Pair-wise comparisons between SSD individuals and healthy controls for scores from individual BACS subtests and composite scores were performed to evaluate the validity of German BACS. In order to maintain sensitivity in detecting potential group differences, no correction was applied to *p*-values subsequent to multiple comparisons. However, the majority of the comparisons had p-values which were considerably smaller than the alpha level (0 = 0.05) and would have survived a correction procedure. Furthermore, diagnostic accuracy was assessed as another measure of validity using receiver operating characteristics (ROC) calculated based on sensitivity and specificity derived from BACS composite scores, where SSD patients were declared as cases and healthy individuals as non-cases. Finally, the German BACS subtests and composite scores were correlated with PANSS total scores.

Internal consistency of the German BACS data was assessed as the measure of reliability. McDonald's Omega was calculated in this study to determine the internal consistency of the German BACS data structure. Here, a correlation matrix was first calculated to examine whether our BACS subtests were suitable for this analysis. Upon that an exploratory factor analysis with oblique rotation was initially performed, determining the number of factors in our BACS data using a scree plot. McDonald's Omega was then calculated for the outcome of the factor analysis. For better comparability, we also reported Cronbach's Alpha, since it has been used in most of the previous studies examining internal consistency of the BACS (Kaneda et al., 2007; Mazhari et al., 2014; Araújo et al., 2015). Furthermore, principal component analysis was performed to further elucidate the number of factors explaining the variance in BACS data. To enhance the evaluation of the psychometric quality, relevant items of the COSMIN Risk of Bias Checklist were assessed and the completed checklist is provided in the supplementary material (Mokkink et al., 2024).

All calculations and statistical analysis were performed in R 4.3.2 (R Core Team, 2024). The level of statistical significance was set at *p* < 0.05 for all tests and comparisons.

## 3 Results

### 3.1 Normative data

The final sample included 107 individuals diagnosed with SSD (39 % females, age: 39.70 ± 11.42 years, range = 18–63, 93.5 % with schizophrenia or schizoaffective disorder, see supplementary table 1) and 175 healthy controls (54 % females, age: 33.95 ± 12.65 years, range = 18–65). Overall, *n* = 175 healthy individuals and *n* = 105 individuals with SSD completed all six subtests of the German BACs and a composite score was calculated for all of them, which constitutes a normative sample for the German BACS. *N* = 92 individuals with SSD were defined as “clinically stable” (59 % females, age: 39.77 ± 11.36, range = 18–63) and formed a replication subgroup. *N* = 38 individuals with SSD (24 % females) and *N* = 111 healthy subjects (53 % females) were between 18 and 35 years old.

Table 2 reports the number of subjects who concluded individual subtests of BACs along with means, standard deviations, and z-scores for each subtest and the BACS composite score for SSD patients and healthy subjects.

**Table 2:** Descriptive statistics of German BACS subtests and the composite score for both SSD cohorts and healthy subjects along with t-statistics and p-values for pair-wise comparisons between each SSD patient group and healthy subjects.

	Healthy subjects			All SSD cohort						Clinically stable SSD cohort					
	N	Mean	SD	N	Mean	SD	z	t	p	N	Mean	SD	z	t	p
VM	175	57.56	7.66	107	47.71	11.61	−1.29	7.63	< 0.001	92	48.21	11.33	−1.22	6.74	< 0.001
VF	175	56.23	13.22	107	47.90	12.96	−0.63	5.07	< 0.001	92	48.82	13.33	−0.56	4.14	< 0.001
SC	175	66.57	12.02	107	50.93	12.23	−1.30	10.66	< 0.001	92	51.84	11.60	−1.23	9.50	< 0.001
DS	175	22.85	3.79	107	19.79	4.51	−0.81	5.83	< 0.001	92	20.01	4.50	−0.75	4.85	< 0.001
TM	175	71.12	11.44	107	58.38	11.31	−1.11	9.30	< 0.001	92	59.04	10.95	−1.06	8.39	< 0.001
TL	175	18.73	2.02	105	17.05	3.03	−0.83	4.93	< 0.001	90	17.14	2.85	−0.78	4.73	< 0.001
CS	175	0	0.64	105	−0.97	0.85	−1.52	10.20	< 0.001	90	−0.90	0.82	−1.41	9.04	< 0.001

**Abbreviations:** VM = Verbal Memory, VF = Verbal Fluency, SC = Symbol Coding, DS = Digit Sequencing, TM = Token Motor, TL = Tower of London, CS = Composite Score.

### 3.2 Group comparisons and diagnostic accuracy

**Fig. 1:**
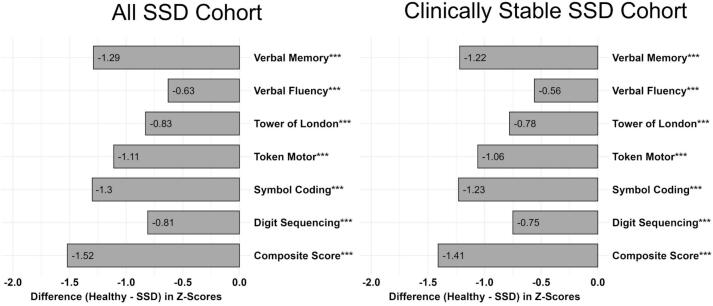
All SSD Cohort and Clinically Stable SSD Cohort had significantly lower composite scores and performance on all subtests of the BACS when compared to healthy controls; (***): *P* < 0.001.

**Fig. 2:**
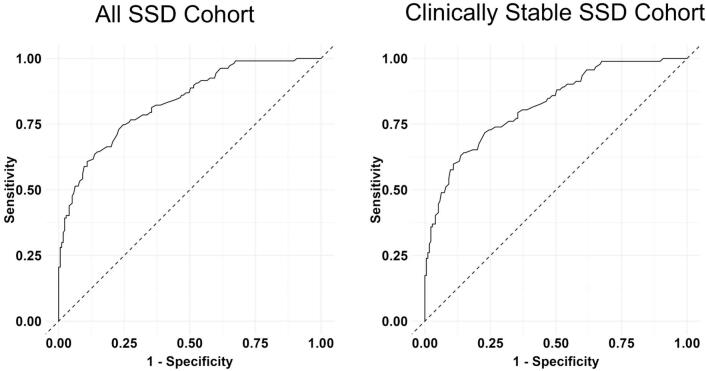
The ROC-curve for the predicted probability for being either identified as being in All SSD Cohort (cases, AUC = 0.83, 95 % CI: 0.78–0.88) and Clinically Stable SSD Cohort (cases, AUC = 0.82, 95 % CI: 0.77–0.87), or healthy subjects group (non-cases).

Fig. 1 shows the average z-scores for all and clinically stable SSD patient groups across the different subtests and the composite score. Table 2 includes the individual t-statistics and *p*-values for all subtests and the composite scores. All z-scores were lower when compared to the z-score of 0, representing the mean value of healthy subjects, and these findings were statistically significant (Ps < 0.001). Of note, the z-score for BACS composite score in all SSD individuals was −1.51, indicating cognitive performance lower than 1.5 standard deviation compared to the average of healthy subjects. Among BACS subsets, the symbol coding exhibited the poorest performance (z = −1.21) in individuals with SSD, followed by verbal memory and token motor task, all of which demonstrated performance levels that were more than one standard deviation below the mean of healthy subjects (z-scores < −1). The BACS subset showing the smallest difference between SSD individuals and healthy subjects was the verbal fluency test (z = 0.63). Furthermore, the ROC analysis of diagnostic accuracy for all patients group demonstrated an area under the curve (AUC) of 0.83 (95 % CI: 0.78, 0.88 with sensitivity and specificity of 0.75 and 0.75, respectively (Fig. 2). For the clinically stable patients, the ROC-curve showed an AUC of 0.82 (95 % CI: 0.77, 0.87) with sensitivity and specificity measures of 0.63 and 0.86, respectively (Fig. 2). The ROC metrics indicated a good degree of differentiation between healthy subjects and both SSD patient groups for the model when applied to BACS composite scores. The sub-sample including younger SSD individuals and healthy subjects, with the age range of 18–35 years, corresponding to that of Sachs et al. (2014), had significantly negative z-scores for BACS subsets and composite score (Ps < 0.001, average BACS composite z-score = −1.41), except for Tower of London test which showed a negative z-score (*P* = 0.058). ROC-curve revealed a good degree of differentiation between younger SSD and healthy individuals, with an AUC of 084 (95 % CI: 0.77, 0.92), sensitivity of 0.82, and specificity of 0.76. The BACS subsets and composite z-scores were also calculated and compared between SSD individuals and healthy subjects once again separately for males and females, yielding similar results to that all SSD individuals described previously for both sexes (see Supplementary table 2 for more details).

### 3.3 BACS subtests and PANSS associations

Tables 3 shows correlations matrices between scores of all BACS subtests. There was a significant correlation (Pearson r coefficients >0.13, *Ps* < 0.01) between scores of all BACS subtests and between subtests and BACS composite scores for both SSD patient groups and healthy subjects. Consistent with the outcomes observed by Sachs et al. (2011), the magnitude of correlations was slightly stronger in the patient groups, but the general correlation pattern of correlations was similar across all groups. Furthermore, a higher BACS composite score was significantly correlated with lower PANSS positive, negative and total scores in all SSD individuals (Pearson r coefficients < −0.2, Ps < 0.05).

**Table 3:** Correlation matrix between BACS measures for healthy controls and both SSD cohorts.

Variable	VMz	DSz	TMz	VFz	SCz	TLz	CSz
VMz	–	0.32**	0.19*	0.33**	0.48**	0.26**	0.67**
DSz	0.54**	–	0.16*	0.36**	0.29**	0.34**	0.64**
TMz	0.32**	0.3**	–	0.13	0.32**	0.34**	0.55**
VFz	0.47**	0.58**	0.24*	–	0.44**	0.15	0.62**
SCz	0.52**	0.54**	0.47**	0.53**	–	0.32**	0.74**
TLz	0.33**	0.46**	0.43**	0.35**	0.51**	–	0.62**
CSz	0.75**	0.78**	0.58**	0.68**	0.79**	0.74**	–

**Legend and abbreviations:** Healthy controls (above diagonal), all patients group (below diagonal). Stars indicate levels of significance: *p < 0.05, **p < 0.01. VM = Verbal Memory, VF = Verbal Fluency, SC = Symbol Coding, DS = Digit Sequencing, Tm = Token Motor, Tl = Tower of London, CS = Composite Score.

### 3.4 Internal consistency and data structure

The exploratory factor analysis after oblique rotation revealed a single factor data structure for our BACS data. McDonald's omega showed a robust overall reliability score (ω = 0.72), marginally exceeding that of Cronbach's alpha (α = 0.71). The congruence of the hierarchical omega value (ω_h = 0.72) with the total omega indicates a unidimensional data structure for the BACS data. This was corroborated by results from the principal component analysis, where the first component accounted for 41.6 % of the total variance. A second component explained a further 16.9 % of the variance, but the eigenvalue associated with the first component (=2.50) signified its predominant role in explaining the variance observed in the BACS data structure compared to the second component, which had a lower, yet significant, eigenvalue (=1.01). Together with a scree plot (Supplementary Fig. 1), and as shown previously by the congruence between McDonald's hierarchical omega value and the total omega, a one-dimensional structure appears to be the best fit for our data.

## 4 Discussion

Using the largest available case-control cognitive dataset to date in German-speaking people with SSD, this study assessed the psychometric properties of the German version of BACS battery, which had been previously evaluated in a preliminary study using a small non-representative sample (Sachs et al. (Sachs et al., 2011)). The mean scores for all BACS subsets and the composite score were found to be significantly lower than the average score for healthy subjects (BACS composite z-score = −1.51). The ROC demonstrated that the BACS exhibited notable discriminatory capacity between individuals with SSD and controls (AUC = 0.83), with favorable sensitivity and specificity indices. Moreover, a higher BACS composite score was linked to better performance on all BACS subtests and lower symptom severity in individuals with SSD. The high internal consistency of the German BACS was confirmed by a single factor data structure, which was determined to be the optimal fit for the data set. As demonstrated by a plethora of prior literature, the evidence presented in this study regarding global cognitive impairment in patients with SSD, as well as in specific cognitive domains, is well-established and not a novelty of this report. Notwithstanding, the present study employed a large cohort of individuals with SSD and healthy subjects to establish the validity and reliability of the German version of BACS. Moreover, our study provides a representative normative sample of German BACS data for future studies in German-speaking populations.

Our findings corroborate those of Sachs et al. (Sachs et al., 2011), indicating that individuals with SSD exhibit diminished cognitive performance across all domains of the German BACS when compared to healthy subjects. The BACS composite score for individuals with SSD was 1.5 standard deviations lower than the mean composite score for healthy subjects. This finding is consistent with the current understanding of cognitive impairment in SSD as outlined in the state-of-the-art literature (McCutcheon et al., 2023; Keefe et al., 2011; Fatouros-Bergman et al., 2014; August et al., 2012). The performance of the subjects in all BACS domains, except for verbal fluency, was approximately one standard deviation below the mean performance of healthy subjects. The lowest outcomes were observed in the domains of symbol coding, verbal memory, and token motor. This consistently low performance across all cognitive domains suggests a global impairment of cognitive functions in SSD, affecting either parent cognitive domains such as processing speed and attention (Burton et al., 2013; Knowles et al., 2015; Nuechterlein et al., 2004) or basic cognitive capacities like reaction times (Nuechterlein et al., 2004).

Similar to our own findings, Sachs and colleagues (Sachs et al., 2011) also demonstrated significant correlations between all BACS subtests and the BACS composite score, except for the token motor task. The larger sample size in the current study enhanced the power of the statistical analysis and replicated the patterns previously reported by Sachs and colleagues (Sachs et al., 2011) for the German version of BACS. The results of the ROC analysis indicated favorable diagnostic accuracy for the BACS composite score, with satisfactory sensitivity and specificity indices. These findings replicate those of Sachs et al. (Sachs et al., 2011) Our findings regarding lower BACS performance in SSD individuals compared to controls, in addition to the favorable diagnostic accuracy of BACS composite scores, were replicated in our subsamples, comprising only clinically stable patients, males and females, and younger SSD individuals (age 18–35 years). This observation in our dataset ensures the general validity of the German version of BACS across the full age range, in males and females, and across the spectrum of clinical severities.

Our findings on the factor structure indicate that a single component provides a more comprehensive explanation of the variance in BACS data, with a second component having a supportive but a less substantial role. Our findings are consistent with those of previous studies (Mazhari et al., 2014; Araújo et al., 2015), who also reported a one-factor solution to be the optimal fit for their BACS data. However, our results differ from those of Keefe et al. (Keefe et al., 2004), who reported a three-component solution to explain the variance in BACS data. Our findings do not resolve the variability observed in previous reports and suggest that there may be a singular underlying construct of cognitive function for the German version of the BACS. Furthermore, the exploratory factor analysis indicated that the BACS dataset exhibited a single-factor structure. The McDonald's omega and Cronbach's alpha coefficients yielded comparable results for internal consistency and uniformity for the German BACS dataset. This finding is consistent with adaptations of the BACS in Brazilian Portuguese (Araújo et al., 2015), Japanese (Kaneda et al., 2007), and Persian (Mazhari et al., 2014) contexts, where Cronbach's alpha demonstrated comparable reliability to the present findings. The congruence between the hierarchical omega and the total omega in our study serves to reinforce the unidimensional nature of the German BACS data, thereby indicating a substantial degree of internal consistency and reliability for the German version of the BACS.

The considerable heterogeneity of clinical phenotypes within SSD precludes the generalization of the findings of this study to the entire disease spectrum. Furthermore, our control sample was on average younger with a higher proportion of females compared to our SSD sample which might have influenced our results. The reported higher education in individuals with SSD compared to healthy controls should be interpreted with caution since this difference was strongly influenced by the calculation of education itself which differs greatly across countries and by an outlier in the SSD group. Further studies are required to accurately represent all diagnostic subgroups of SSD. Furthermore, the cross-sectional nature of our data prevents us from conducting a longitudinal investigation of psychometric properties, such as test-retest reliability, which has been previously examined by Sachs et al. (Sachs et al., 2011) It is recommended that future studies employ multiple testing sessions, spaced appropriately spaced apart to minimize confounding effects of practice or training. Finally, an important limitation of the present study is the lack of direct assessment of criterion-related validity, as we did not correlate BACS scores with other established cognitive assessment tools. Future research should evaluate the concurrent validity of the German BACS by examining its associations with other standardized measures of cognitive functioning, such as the MATRICS Consensus Cognitive Battery or similar validated instruments. Establishing such correlations would further strengthen the evidence for the criterion-related validity of the German BACS.

## 5 Conclusion

In conclusion, this study substantiated the validity and reliability of the German version of BACS in the largest cohort of SSD patients and healthy controls so far. The findings corroborated the cognitive impairment of individuals with SSD, as their cognitive performance fell below 1.5 standard deviations from the mean of healthy subjects, in accordance with the current state of the literature. Our dataset provides a representative normative sample of BACS outcomes for research on SSD in German-speaking populations.

## CRediT authorship contribution statement

**Matin Mortazavi:** Writing – review & editing, Writing – original draft, Visualization, Supervision, Software, Methodology, Investigation, Formal analysis, Conceptualization. **Jakob Amon:** Writing – original draft, Visualization, Methodology, Formal analysis. **Iris Jäger:** Formal analysis, Data curation. **Genc Hasanaj:** Writing – review & editing, Conceptualization. **Zahra Aminifarsani:** Writing – review & editing, Formal analysis. **Kristin Fischer:** Writing – review & editing. **Matthias Gamer:** Writing – review & editing, Supervision, Formal analysis. **Alkomiet Hasan:** Writing – review & editing, Writing – original draft, Supervision, Project administration, Investigation, Funding acquisition, Data curation, Conceptualization. **Richard S.E. Keefe:** Writing – original draft. **Gabriele Sachs:** Writing – original draft. **Peter Falkai:** Investigation, Funding acquisition, Conceptualization. **Daniel Keeser:** Writing – review & editing, Investigation, Funding acquisition, Data curation, Conceptualization. **Florian Raabe:** Writing – review & editing, Methodology, Investigation, Funding acquisition, Data curation, Conceptualization. **Elias Wagner:** Writing – review & editing, Writing – original draft, Supervision, Resources, Project administration, Methodology, Investigation, Funding acquisition, Formal analysis, Data curation, Conceptualization.

## Declaration of competing interest

The authors declare that there are no conflicts of interest in relation to the subject of this study. General declaration of potential conflict of interests: AH received speaker fees from AbbVie, Advanz, Janssen, Otsuka, Lundbeck, Rovi, and Recordati and was a member of the advisory boards of these companies and Boehringer-Ingelheim. GS has been a consultant to or has received honoraria from Angelini, Boehringer Ingelheim, Janssen Cilag, Lundbeck, Mylan, Recordati. RSEK was CEO at Verasci, Inc. when these data were collected and now serves as a consultant to WCG, Karuna, Novartis, Kynexis, Gedeon-Richter, Pangea, Merck, and Boehringer-Ingelheim, and receives royalties for the BAC-app, BACS and VRFCAT. EW has been invited to advisory boards by Recordati, Boehringer Ingelheim and Teva. All other authors report no potential conflicts of interest.

## Appendix A Supplementary data

Supplementary material 1 Image 1

Supplementary material 2 Image 2
