# Supplementary material for: Assessment of the German Version of Brief Assessment of Cognition in Schizophrenia (BACS)
Source: Schizophr Res Cogn. 2025 Apr 30;41:100364. doi: 10.1016/j.scog.2025.100364 (PMC12084069; doi:10.1016/j.scog.2025.100364)
Supplement: Supplementary file 1 — Supplementary material 1 [file mmc1.docx]

**
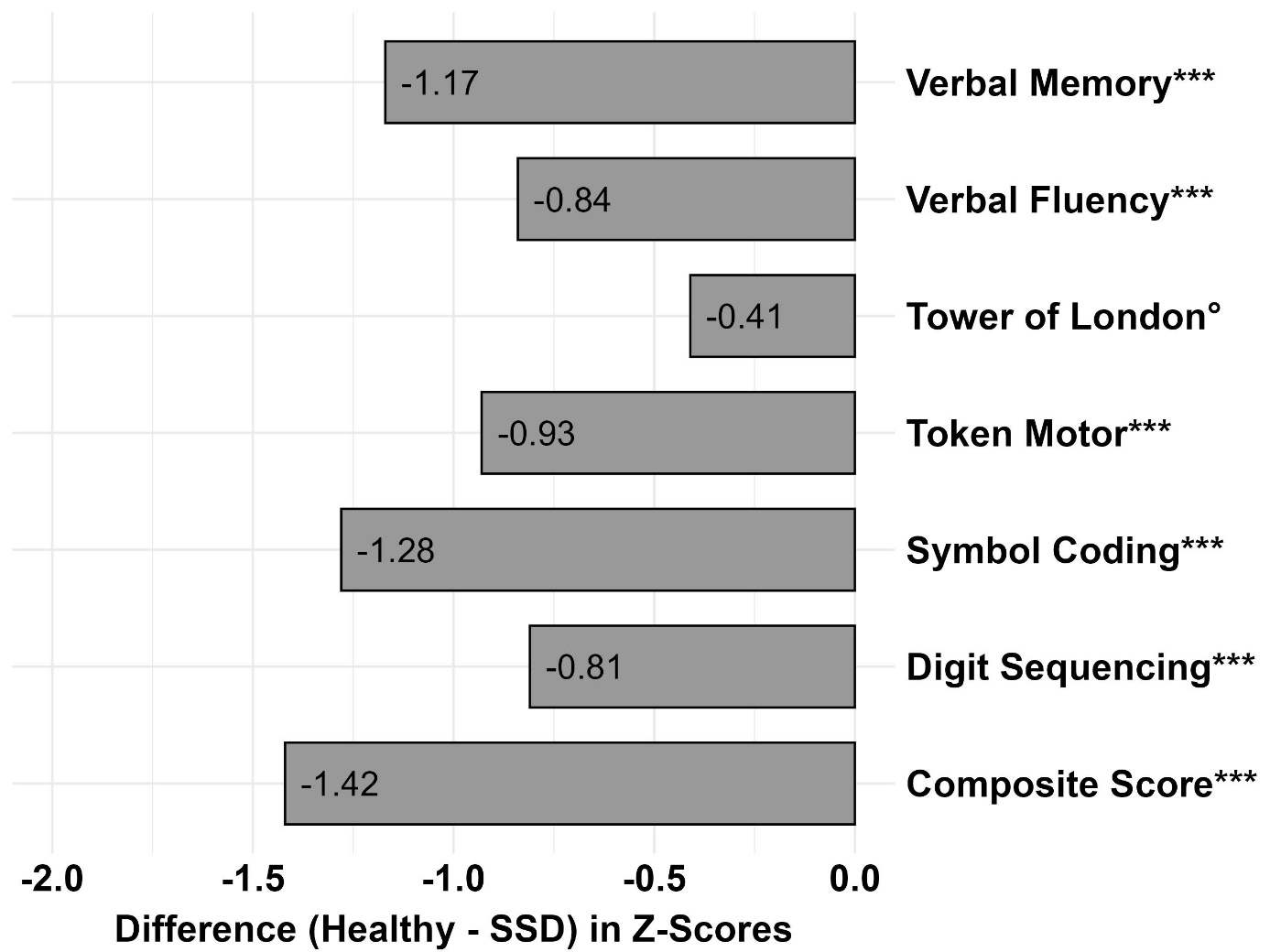
**

**Supplementary Figure 1.** Individuals in the younger SSD cohort (age range: 18-35 years old) had significantly lower composite scores and performance on all subtests of the BACS when compared to healthy controls, (***) : P < 0.001; (°): P < 0.1.


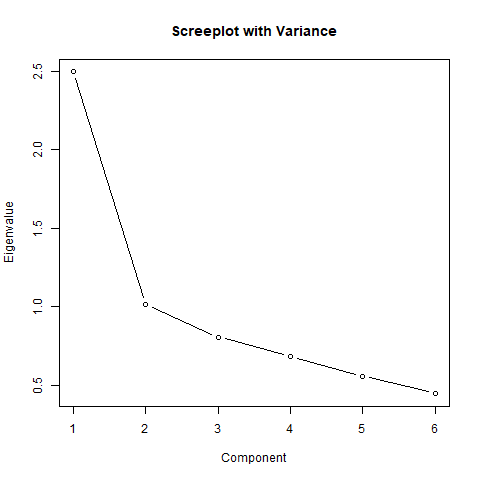


**Supplementary Figure 2**. Scree plot showing the eigenvalues calculated by the principal component analysis. Components 1 and 2 had significant contributions in explaining the variance, with component 1 having a more substantial role.

**Supplementary table 1.** Distribution of SSD diagnosis in All SSD Cohort according to Mini International Neuropsychiatric Interview (MINI, version 7.0.02, based on DSM 5).

|  | N | Female/Male |
| --- | --- | --- |
| Schizophrenia | 70 | 20/50 |
| Schizoaffective disorder | 30 | 18/12 |
| Brief Psychotic Episode | 3 | 1/3 |
| Delusion Disorder | 1 | 1/0 |
| Drug induced Psychosis | 0 | 0/0 |
| Unspecified Schizophrenia/Psychotic Disorder | 3 | 0/3 |

**Supplementary table 2.** Descriptive statistics of German BACS subtests and the composite score for Male and Female All SSD cohorts along with t-statistics and p-values for pair-wise comparisons between each SSD patient group and the corresponding Male and Female healthy subjects.

| Male All SSD Cohort | | | | | | | Female All SSD Cohort | | | |
| --- | --- | --- | --- | --- | --- | --- | --- | --- | --- | --- |
|  | N | z | t |  | p |  | N | z | t | p |
| VM | 65 | -0.92 | 4.49 |  | < 0.001 |  | 42 | -1.80 | 5.81 | < 0.001 |
| VF | 65 | -0.47 | 2.92 |  | 0.004 |  | 42 | -0.82 | 4.31 | < 0.001 |
| SC | 65 | -1.17 | 6.86 |  | < 0.001 |  | 42 | -1.44 | 7.80 | < 0.001 |
| DS | 65 | -1.01 | 5.21 |  | < 0.001 |  | 42 | -0.66 | 3.21 | 0.002 |
| TM | 65 | -0.91 | 5.71 |  | < 0.001 |  | 42 | -1.35 | 7.09 | < 0.001 |
| TL | 65 | -0.72 | 3.37 |  | 0.001 |  | 42 | -1.05 | 3.99 | < 0.001 |
| CS | 64 | -1.29 | 6.70 |  | < 0.001 |  | 42 | -1.83 | 7.23 | < 0.001 |

**Abbreviations:** VM = Verbal Memory, VF = Verbal Fluency, SC = Symbol Coding, DS = Digit Sequencing, TM = Token Motor, TL = Tower of London, CS = Composite Score

**Supplementary table 3.** Correlation matrix among BACS measures for healthy controls and the clinically stable SSD cohort.

| Variable | VMz | DSz | TMz | VFz | SCz | TLz | CSz |
| --- | --- | --- | --- | --- | --- | --- | --- |
| VMz | - | 0.32** | 0.19* | 0.33** | 0.48** | 0.26** | 0.67** |
| DSz | 0.53** | - | 0.16* | 0.36** | 0.29** | 0.34** | 0.64** |
| TMz | 0.25* | 0.30** | - | 0.13 | 0.32** | 0.34** | 0.55** |
| VFz | 0.5** | 0.56** | 0.23* | - | 0.44** | 0.15 | 0.62** |
| SCz | 0.46** | 0.54** | 0.4** | 0.54** | - | 0.32** | 0.74** |
| TLz | 0.31** | 0.45** | 0.43** | 0.37** | 0.49** | - | 0.62** |
| CSz | 0.72** | 0.78** | 0.54** | 0.7** | 0.75** | 0.74** | - |

**Legend and abbreviations:** Healthy controls (above diagonal), clinically stable patients group (below diagonal). Stars indicate levels of significance: **p* < .05, ***p* < .01. VM = Verbal Memory, VF = Verbal Fluency, SC = Symbol Coding, DS = Digit Sequencing, Tm = Token Motor, Tl = Tower of London, CS = Composite Score.
