# Supplementary material for: Assessment of the German Version of Brief Assessment of Cognition in Schizophrenia (BACS)
Source: Schizophr Res Cogn. 2025 Apr 30;41:100364. doi: 10.1016/j.scog.2025.100364 (PMC12084069; doi:10.1016/j.scog.2025.100364)
Supplement: Supplementary file 2 — Supplementary material 2 [file mmc2.pdf]

Box 3. Structural validity

Statistical methods	very good	adequate	doubtful	inadequate	NA
1 For CTT: Was exploratory or confirmatory factor analysis performed?	Confirmatory factor analysis performed	Exploratory factor analysis performed	Only PCA was performed	No exploratory or confirmatory factor analysis performed	NA
2 For IRT/Rasch: does the chosen model fit to the research question?	Chosen model fits well to the research question	Assumable that the chosen model fits well to the research question	Doubtful if the chosen model fits well to the research question	Chosen model does not fit to the research question	NA
3 Was the sample size included in the analysis adequate?	FA: 7 times the number of items in the tested model and ≥100	FA: at least 5 times the number of items in the tested model and ≥100; OR at least 6 times number of items in the tested model but <100	FA: 5 times the number of items in the tested model but <100	FA: < 5 times the number of items in the tested model	
	Rasch/1PL models: ≥ 200 subjects	Rasch/1PL models: 100-199 subjects	Rasch/1PL models: 50-99 subjects	Rasch/1PL models: < 50 subjects	
	2PL parametric IRT models OR Mokken scale analysis: ≥ 1000 subjects	2PL parametric IRT models OR Mokken scale analysis: 500-999 subjects	2PL parametric IRT models OR Mokken scale analysis: 250-499 subjects	2PL parametric IRT models OR Mokken scale analysis: < 250 subjects	
Other					
4 Were there any other important flaws in the design or statistical methods of the study?	No other important methodological flaws		Other minor methodological flaws (e.g. rotation method not described)	Other important methodological flaws (e.g. inappropriate rotation method)	

**Box 4. Internal consistency**					
Statistical methods	**very good**	**adequate**	**doubtful**	**inadequate**	**NA**
1 For continuous scores: Was Cronbach's alpha or omega calculated?	Cronbach's alpha, or Omega calculated		Only item-total correlations calculated	No Cronbach's alpha and no item-total correlations calculated	NA
2 For dichotomous scores: Was Cronbach's alpha or KR-20 calculated?	Cronbach's alpha or KR-20 calculated		Only item-total correlations calculated	No Cronbach's alpha or KR-20 and no item-total correlations calculated	NA
3 For IRT-based scores: Was standard error of the theta (SE (θ)) or reliability coefficient of estimated latent trait value (index of (subject or item) separation) calculated?	SE(θ) or reliability coefficient calculated			SE(θ) or reliability coefficient NOT calculated	NA
Other					
4 Were there any other important flaws in the design or statistical methods of the study?	No other important methodological flaws		Other minor methodological flaws	Other important methodological flaws	

**9b. Comparison between subgroups (discriminative or known-groups validity)**					
Design requirements	**very good**	**adequate**	**doubtful**	**inadequate**	**NA**
5 Was an adequate description provided of important characteristics of the subgroups?	Adequate description of the important characteristics of the subgroups	Adequate description of most of the important characteristics of the subgroups	Poor of no description of the important characteristics of the subgroups		
Statistical methods					
6 Were statistical methods appropriate for the subgroups being compared?	Statistical methods applied were appropriate	Assumable that statistical methods were appropriate	Statistical methods applied NOT optimal	Statistical methods applied NOT appropriate	
Other					
7 Were there any other important flaws in the design or statistical methods of the study?	No other important methodological flaws		Other minor methodological flaws	Other important methodological flaws	

Repeated measurements were not performed in this study and thus test-retest reliability could not be examined. This has been mentioned as a limitation in the discussion.

**Box 6. Reliability**					
Design requirements	**very good**	**adequate**	**doubtful**	**inadequate**	**NA**
1 Were patients stable on the construct to be measured in the time between the repeated measurements?	Evidence provided that patients were stable	Assumable that patients were stable	Unclear if patients were stable	Patients were NOT stable	
2 Was the time interval between the repeated measurements appropriate?	Time interval appropriate		Doubtful if time interval was appropriate OR time interval was not stated	Time interval NOT appropriate	
3 Were the measurement conditions similar for the repeated measurements – except for the condition being evaluated?	Measurement conditions were similar (evidence provided)	Assumable that measurement conditions were similar	Unclear if measurement conditions were similar	Measurement conditions were NOT similar	
Statistical methods					
4 For continuous scores: Was the appropriate intraclass correlation coefficient (ICC) calculated?	$ICC_{agreement}$ was or can be calculated	($ICC_{consistency}$, Pearson or Spearman correlation coefficient was calculated, OR the ICC model or formula was not described)<br><br>WITH evidence provided that no systematic change between measurements has occurred<br><br>OR $ICC_{one\text{-}way}$ was calculated	($ICC_{consistency}$, Pearson or Spearman correlation coefficient was calculated OR the ICC model or formula was not described)<br><br>WITHOUT evidence provided that no systematic change between measurements has occurred	($ICC_{consistency}$, Pearson or Spearman correlation coefficient was calculated OR the ICC model or formula was not described)<br><br>WITH evidence provided that a systematic change between measurements has occurred	NA

		very good	adequate	doubtful	inadequate	NA
5	For dichotomous scores: was kappa calculated?	Kappa calculated				NA
6	For nominal scores: was an unweighted kappa calculated?	Unweighted kappa calculated				NA
7	For ordinal scores: was a weighted kappa calculated?	Weighted kappa calculated and the weighting scheme was described	Kappa calculated, but weighting scheme not described	Unweighted Kappa calculated or unclear if weighting was applied		NA
Other						
8	Were there any other important flaws in the design or statistical methods of the study?	No other important methodological flaws		Other minor methodological flaws	Other important methodological flaws	

Box 7. Measurement error

Design requirements		very good	adequate	doubtful	Inadequate	NA
1	Were patients stable on the construct to be measured in the time between the repeated measurements?	Evidence provided that patients were stable	Assumable that patients were stable	Unclear if patients were stable	Patients were NOT stable	
2	Was the time interval between the repeated measurements appropriate?	Time interval appropriate		Doubtful if time interval was appropriate OR time interval was not stated	Time interval NOT appropriate	
3	Were the measurement conditions similar for the measurements – except for the condition being evaluated as a source of variation?	Measurement conditions were similar (evidence provided)	Assumable that measurement conditions were similar	Unclear if measurement conditions were similar	Measurement conditions were NOT similar	

Statistical methods						
4	For continuous scores: was the Standard Error of Measurement (SEM), Smallest Detectable Change (SDC) or Limits of Agreement (LoA) calculated?	$SEM_{agreement}$, $SEM_{one-way}$, $SDC_{agreement}$, or $SDC_{one-way}$ was or could be calculated	($SEM_{consistency}$ or $SDC_{consistency}$ or LoA was calculated OR the SEM/SDC model or formula was not described)<br><br>WITH evidence provided that no systematic change between the measurement has occurred	($SEM_{consistency,}$ $SDC_{consistency,}$ or LoA was calculated)<br><br>WITHOUT evidence provided that no systematic change between measurements has occurred	SEM calculated based on Cronbach's alpha or SD from another population<br><br>OR<br><br>($SEM_{consistency}$ $SDC_{consistency}$ or LoA calculated)<br>WITH evidence provided that systematic change has occurred	NA
5	For dichotomous/nominal/ordinal scores: was the percentage (positive and negative) agreement calculated?	% positive and negative agreement calculated	% agreement calculated			NA
Other						
6	Were there any other important flaws in the design or statistical methods of the study?	No other important methodological flaws		Other minor methodological flaws	Other important methodological flaws	

Criterion-validity was not assessed in this study, as no other cognitive battery without BACS was used to measure cognition. This limitation has been discussed in the manuscript. Please see the discussion section.

**Box 8. Criterion validity**		**very good**	**adequate**	**doubtful**	**inadequate**	**NA**
Statistical methods						
1	For continuous scores: were correlations, or the AUC calculated?	Correlations or AUC calculated				NA
2	For dichotomous scores: were sensitivity and specificity determined?	Sensitivity and specificity calculated				NA
Other						
3	Were there any other important flaws in the design or statistical methods of the study?	No other important methodological flaws		Other minor methodological flaws	Other important methodological flaws	
